# High-nitrogen fertilizer alleviated adverse effects of drought stress on the growth and photosynthetic characteristics of *Hosta* ‘Guacamole’

**DOI:** 10.1186/s12870-024-04929-5

**Published:** 2024-04-18

**Authors:** Jiao Zhu, Youming Cai, Xin Li, Liuyan Yang, Yongchun Zhang

**Affiliations:** Forest and fruit tree Research Institute, Shanghai Academy of Agricultural Sciences, Shanghai, 201403 China

**Keywords:** *Hosta* ‘Guacamole’, Drought stress, Photosynthetic characteristics, Chlorophyll fluorescence, OEC activity of reaction center

## Abstract

**Background:**

Several plants are facing drought stress due to climate change in recent years. In this study, we aimed to explore the effect of varying watering frequency on the growth and photosynthetic characteristics of *Hosta* ‘Guacamole’. Moreover, we investigated the effect of high-nitrogen and -potassium fertilizers on alleviating the impacts of drought stress on the morphology, photosynthetic characteristics, chlorophyll fluorescence, fast chlorophyll a fluorescence transient, JIP-test parameters, and enzymatic and non-enzymatic scavenging system for reactive oxygen species (ROS) in this species.

**Results:**

Leaf senescence, decreased chlorophyll contents, limited leaf area, and reduced photosynthetic characteristics and oxygen-evolving complex (OEC) activity were observed in *Hosta* ‘Guacamole’ under drought stress. However, high-nitrogen fertilizer (30-10-10) could efficiently alleviate and prevent the adverse effects of drought stress. High-nitrogen fertilizer significantly increased chlorophyll contents, which was higher by 106% than drought stress. Additionally, high-nitrogen fertilizer significantly improved net photosynthetic rate and water use efficiency, which were higher by 467% and 2900% than those under drought stress. It attributes that high-nitrogen fertilizer could reduce transpiration rate of leaf cells and stomatal opening size in drought stress. On the other hand, high-nitrogen fertilizer enhanced actual photochemical efficiency of PS II and photochemical quenching coefficient, and actual photochemical efficiency of PS II significantly higher by 177% than that under drought stress. Furthermore, high-nitrogen fertilizer significantly activated OEC and ascorbate peroxidase activities, and enhanced the performance of photosystem II and photosynthetic capacity compared with high-potassium fertilizers (15-10-30).

**Conclusions:**

High-nitrogen fertilizer (30-10-10) could efficiently alleviate the adverse effects of drought stress in *Hosta* ‘Guacamole’ via enhancing OEC activity and photosynthetic performance and stimulating enzymatic ROS scavenging system.

**Supplementary Information:**

The online version contains supplementary material available at 10.1186/s12870-024-04929-5.

## Background

Water scarcity is one of the main consequences of climate change caused by global warming and has created abiotic stress environment for a huge number of plants [1]. Drought stress affects plant physiology and metabolism and negatively affects plant growth, stomatal conductance, gas exchange, chlorophyll content, leaf water potential, and photosynthesis [2–4]. Drought in summer often results in the morphological changes in plants such as yellowing, curling, and wilting of leaves and reduces the yield and ornamental value of commercial crops and ornamental plants [5, 6].

*Hosta* species (family: Liliaceae) are herbaceous perennials, which are commercially grown on a large scale for landscaping and for extracting essential oil in many countries [7]. Various members of *Hosta* spp. have varying sizes, from miniature to giant, and they are native to China, Japan, and Korea. Most endemic species are used as edible vegetables and folk medicines [6]. Previous studies reported that *Hosta* spp. is rich in steroids and flavonoids with valuable medicinal properties such as anti-inflammatory, analgesic, antioxidant, antitumor, antiviral, acetylcholinesterase inhibitory, antimicrobial, and anti-chronic-prostatitis [8]. Moreover, flowers of several members of this species have attractive fragrance, with the main aromatic compound being terpenoids (mainly myrcene, limonene, beta-ocimene, and linalool) [9, 10]. Compared with the cultivar, the most characteristic aromatic compound in wild-type *Hosta* flowers was reported to be hexanol. The essential oil obtained from wild-type *H. sieboldiana* flowers was richer in aromatic compounds than that obtained from the cultivar flowers, highlighting the use of wild-type flowers for essential oil extraction [11]. In addition, it is considered a decorative plant because of delicate flowers and because its leaves are of varying sizes, shapes, and colors [12]. *Hosta* spp. has a high ornamental value in gardening industry. The flowers of most members of *Hosta* spp. exhibit colors in the shades of purple and white. Their leaves may be green, blue, yellow, golden, or white and may have one or multiple colors [12]. For example, *H. plantaginea* is used for landscaping and gardening and is appreciated for its ornamental value with beautiful foliage and flowers, as well as long blooming period [8, 13].

Previous studies revealed that various environmental factors affect the growth and photosynthetic capacity of various members of *Hosta* spp [14–16]. It is essential to conserve water during cultivation and avoid morphological changes that can occur in *Hosta* spp. after being in water-deficient environment for a long time [14]. In the view of severe global climate change, increased temperature in summer and reduced rainfall are being observed in China and globally [17]. Severe environmental stresses such as drought stress would affect photosystem II (PS II) in plants [18]. Previous studies reported that the application of growth hormones, silicon, or selenium or molecular and genomic breeding for drought resistance could alleviate the adverse effects of drought stress [3, 4]. However, they have weak operability, hampering its practical use. However, salicylic acid and nitrogen and potassium fertilizers could also alleviate the effects of drought stress in plants [19, 20]. Therefore, the application of fertilizers perhaps is the most convenient and easy way to resist drought stress.

Breeding fragrant cultivars has become the new trend of modern development of *Hosta* [10]. *Hosta* ‘Guacamole’ is an aromatic and ornamental herb and is a *Hosta* hybrid cultivar. Its white flowers have characteristic fragrance and can be used to extract essential oil. Its leaves lack wax coat; therefore, they are perhaps sensitive to temperature, light, and moisture compared with other members of *Hosta* spp. with wax coat. In this study, we aimed to explore the effect of drought stress on the growth and photosynthetic characteristics of *Hosta* ‘Guacamole’ and to further analyze high-nitrogen fertilizers how to alleviate the adverse effects of drought stress. This study provided an effective method to solve the problem of decreasing yield and ornamental value of *Hosta* spp. under drought and provided the theoretical basis for alleviating the adverse effects of drought stress. In addition, our study provided a basis for the cultivation of *Hosta* spp. in the view of climate change.

## Results

### Effect of high-nitrogen and -potassium fertilizers on the morphological characteristics of *Hosta* ‘Guacamole’ under drought stress

**Fig. 1:**
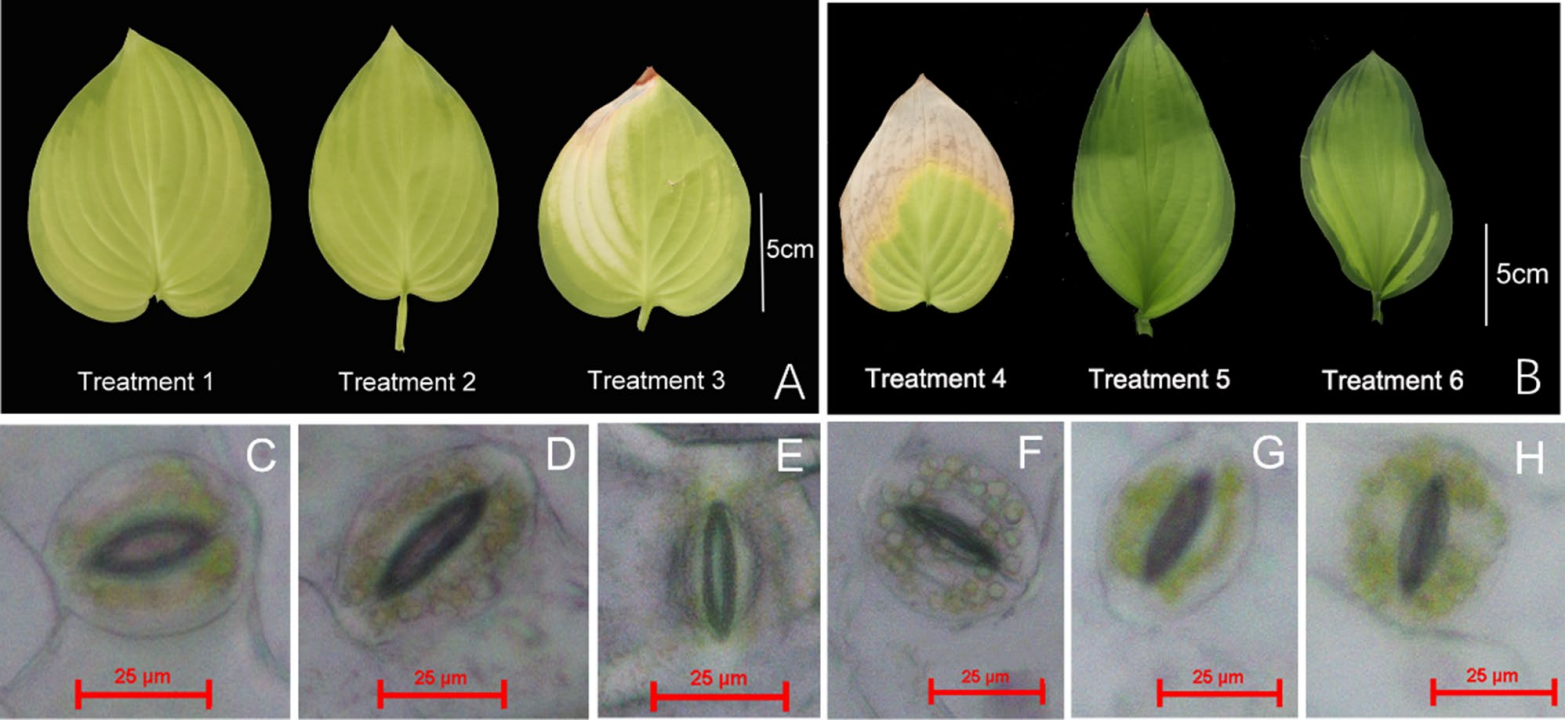
Morphological response in terms of changes in the leaf shape and color under T1-3 (**A**); Morphological response in terms of changes in the leaf shape and color under T4-6 (**B**); stomatal opening under T1 (**C**), T2 (**D**), and T3 (**E**); Stomatal closure under T4 (**F**), T5 (**G**), and T6 (**H**)

The morphology of plants under T3 treatment in drought stress was clearly different. The edges of leaves gradually started turning white (Fig. 1A), and later, the edge of leaves the entire leaf surface turned white under T4 treatment (Fig. 1B). After the fertilizer treatment in T5 and T6, the leaves turned green and had more leaves than T4 (Fig. 1B; Table 1). Under T3, the size of stomatal opening was smaller by 51.8% and 36.3% than that under T1 and T2, respectively (Fig. 1C-E). However, T4, T5, and T6 treatments resulted in smaller and even closure of stomatal opening in longtime drought environment (Fig. 1F-H).

The largest leaf area was under T1, which was higher by 16.7% and 18.7% than that under T2 and T3, respectively. The L^*^ and b^*^ values under T3 treatment were clearly higher by 3.9% and 5.9% than those under T1, respectively. The lowest chlorophyll content was observed under T3, which was lower by 15.6% and 21.0% than that under T1 and T2, respectively. After fertilizer treatment to T3-treated plants, under T5 and T6, the leaf area clearly increased; the largest leaf area was 78.4 ± 5.92^a^ cm^2^ under T5. In addition, under T5 and T6, the chlorophyll content of leaves substantially increased, alleviating the yellowing of the leaves. The highest chlorophyll content was in T5, which was significantly higher 106% than those T4. The L^*^ and b^*^ values were significantly lower and a^*^ value was significantly higher under T5 than under T4 and T6 (Table 1).

**Table 1:** The alleviating effect of high-nitrogen and -potassium fertilizers on the impacts of drought stress on leaf area, color coefficient, and stomatal opening size in *Hosta* ‘Guacamole’

Treatment		T1	T2	T3	T4	T5	T6
Leaf area (cm^2^)		65.4 ± 11.6^a^	56.0 ± 8.66^b^	55.0 ± 6.03^b^	58.2 ± 14.4^c^	78.4 ± 5.92^a^	68.3 ± 8.88^b^
Leaf numbers		3.4 ± 0.61^a^	3.5 ± 0.61^a^	3.5 ± 0.79^a^	4.4 ± 1.07^b^	9.1 ± 2.37^a^	8.5 ± 2.32^a^
Size of stomatal opening (px)		27.6 ± 2.88^a^	20.9 ± 6.95^b^	13.3 ± 3.51^c^	-	-	-
Chlorophyll content (SPAD)		15.4 ± 2.58^a^	16.4 ± 2.17^a^	12.9 ± 1.60^b^	12.3 ± 3.47^b^	25.4 ± 5.63^a^	21.2 ± 5.76^a^
Color coefficient of chromameter	L^*^	60.2 ± 1.66^b^	60.1 ± 1.50^b^	62.6 ± 3.12^a^	59.5 ± 5.50^a^	47.3 ± 6.46^b^	55.2 ± 3.62^a^
	a^*^	−18.1 ± 11.0^a^	−18.4 ± 10.9^a^	−21.8 ± 0.8^a^	−21.4 ± 0.98^a^	−19.6 ± 1.67^a^	−23.7 ± 2.91^b^
	b^*^	46.7 ± 1.29^b^	46.5 ± 1.23^b^	49.5 ± 1.88^a^	46.3 ± 4.07^a^	33.8 ± 7.59^b^	43.9 ± 3.41^a^

Each value is expressed as the mean ± SEof 15 independent plants in T1, T2, and T3, meanwhile 10 independent plants in T4, T5, T6. Different letters indicate significant differences among T1, T2, and T3 treatments or among T4, T5, and T6 treatments (*P* < 0.05)

### Effect of high-nitrogen and -potassium fertilizers on the photosynthetic characteristics of *Hosta* ‘Guacamole’ under drought stress

**Fig. 2:**
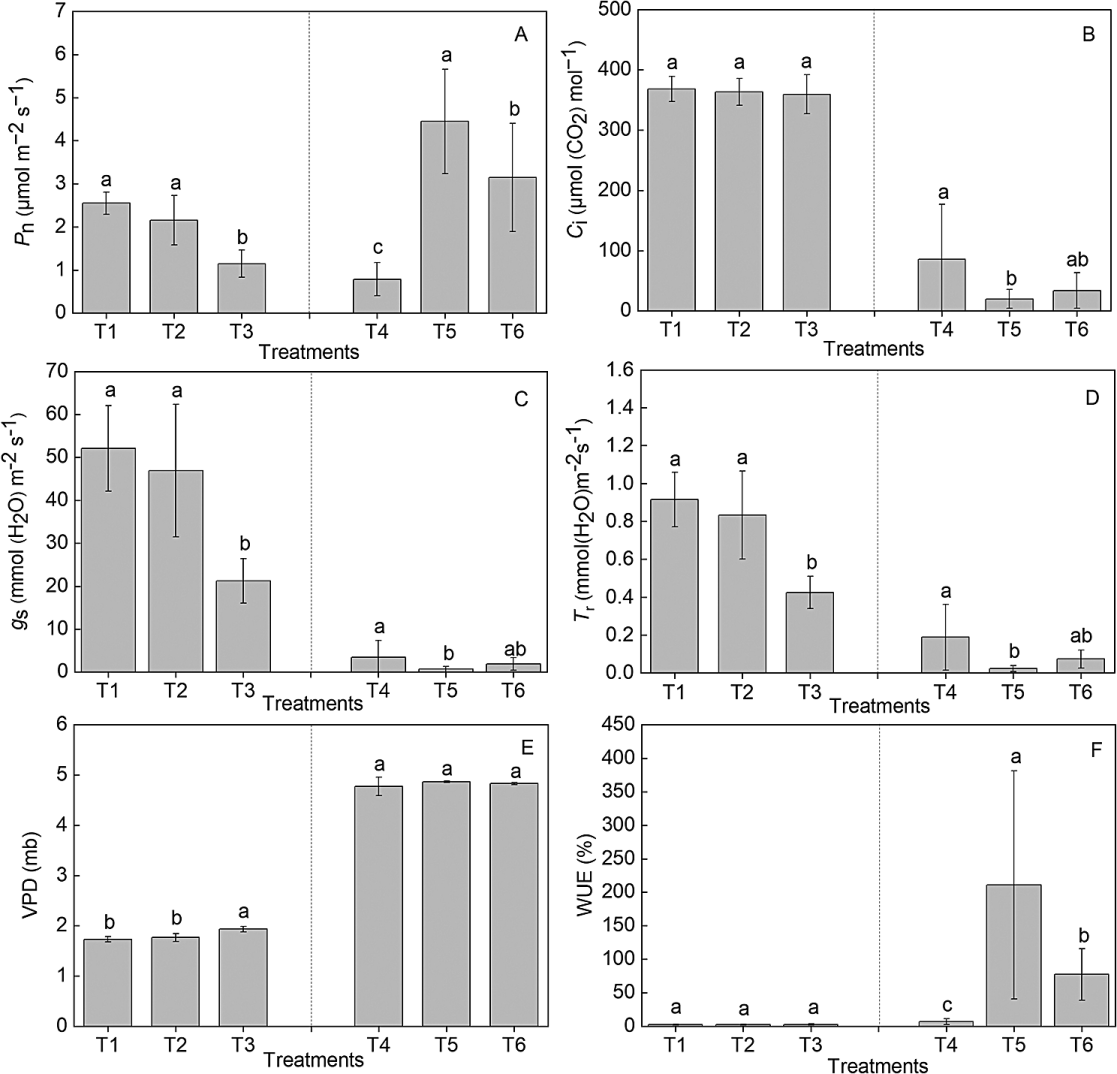
The effect of high-nitrogen and -potassium fertilizers on the *P*_n_ (**A**), *C*_i_ (**B**), *g*_*s*_ (**C**), *T*_r_ (**D**), VPD (**E**), and WUE (**F**) of *Hosta* ‘Guacamole’ under drought stress. Each value is expressed as the mean ± SE of 9 independent plants. Different lowercase letters represent significant differences between treatments (*P* < 0.05)

The maximum net photosynthetic rate (*P*_n_), stomatal conductance (*g*_*s*_), and transpiration rate (*T*_r_) were observed in T1 and T2 compared with T3. However, compared with T1 and T2, T3 treatment exhibited the maximum vapor pressure deficit (VPD). The intercellular CO_2_ concentrations (*C*_i_) and water use efficiency (WUE) were not significantly different among the treatments. After various fertilizer treatments, the *P*_n_ and WUE under T5 were significantly higher than those under T4 and T6. The *P*_n_ under T5 was 4.45 ± 1.20^a^ µmol m^− 2^ s^− 1^, which was significantly higher by 467% than that under T4. However, the *g*_s_, *T*_r_, and *C*_i_ under T4 were significantly higher by 417%, 710%, and 329%, than those under T5, respectively, but no significant difference was observed in terms of VPD (Fig. 2).

### Effect of high-nitrogen and -potassium fertilizers on the chlorophyll fluorescence in *Hosta* ‘Guacamole’ under drought stress

The actual photochemical efficiency of PS II (ΦPS II) and electron transport rate (ETR) were increased as the water content in soil increased. The highest ETR and ΦPS II were observed under T1. The lowest ΦPS II was observed under T3. The lowest ETR and photochemical quenching coefficient (qP) were observed under T3. The highest non-photochemical quenching coefficient (NPQ) was observed under T2 and T3.

**Fig. 3:**
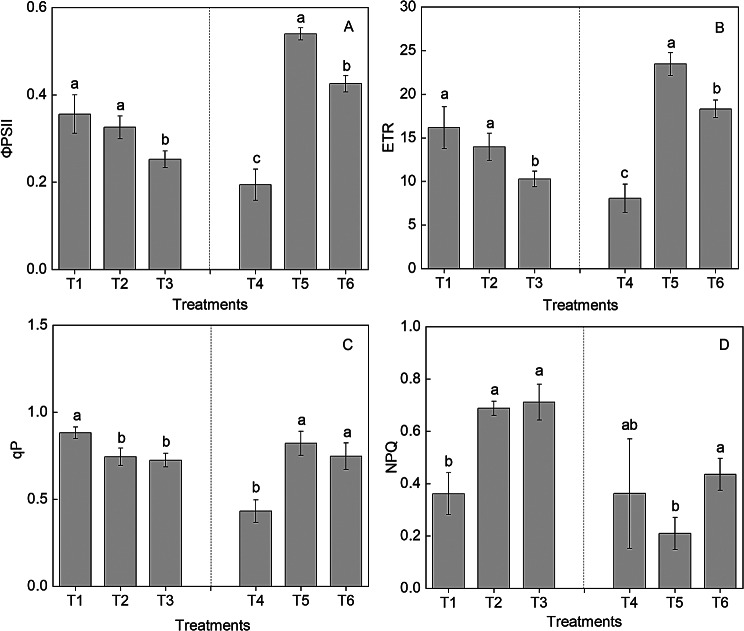
The effect of high-nitrogen and potassium fertilizers on the ΦPS II (**A**), ETR (**B**), qP (**C**), and NPQ (**D**) in *Hosta* ‘Guacamole’ under drought stress. Each value is expressed as the mean ± SE of 3 independent plants, and each treatment was repeated four times. Different lowercase letters represent significant differences between treatments (*P* < 0.05)

After fertilizer treatments, the ΦPS II, ETR, and qP were significantly increased under T5 and T6, exhibiting the maximum value under T5. These values under T5 were 0.539 ± 0.014^a^, 23.4 ± 1.31^a^, and 0.822 ± 0.069^a^, respectively, and were higher by 177%, 191%, and 90.0%, respectively, than those under T4. Conversely, the NPQ exhibited the minimum value under T5 (0.209 ± 0.061^b^), which was significantly lower than that under T4 and T6 (Fig. 3).

### Effect of high-nitrogen and -potassium fertilizers on the chlorophyll fluorescence rise kinetics in *Hosta* ‘Guacamole’ under drought stress

**Fig. 4:**
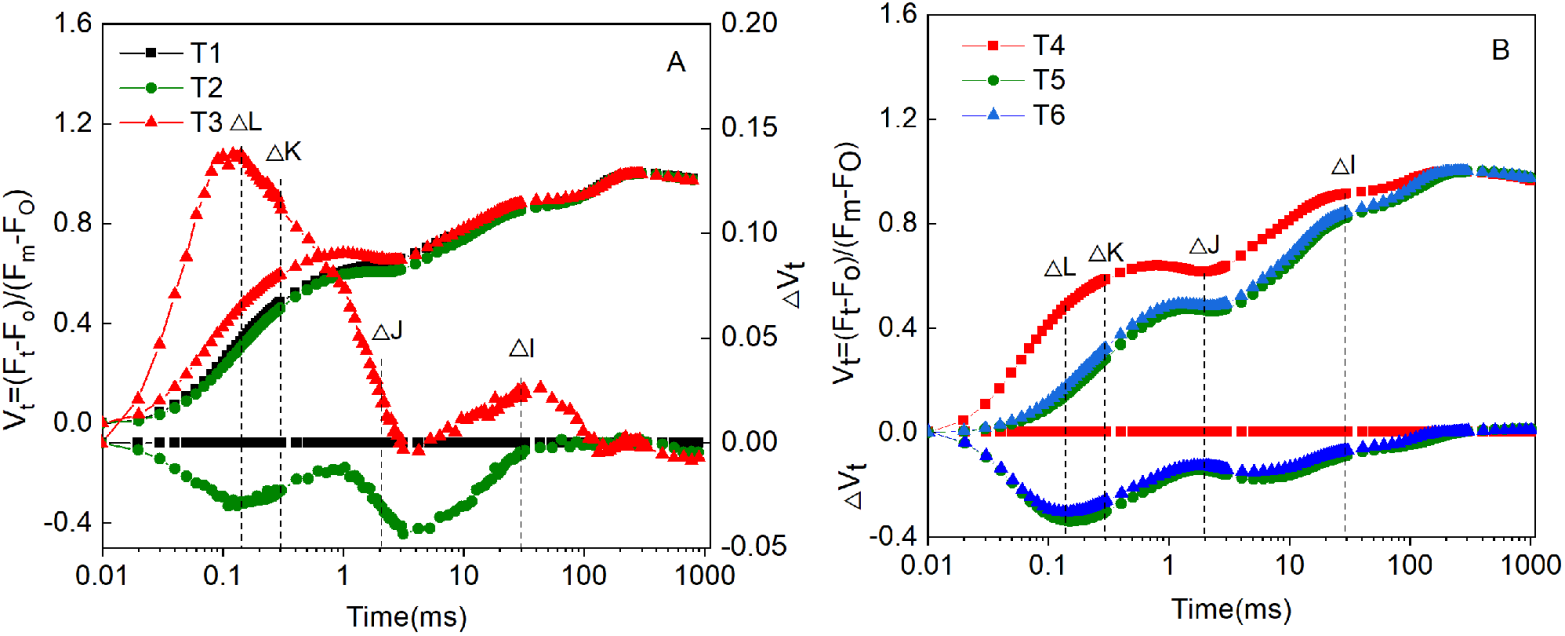
The V_t_ andΔV_t_ in *Hosta* ‘Guacamole’ under T1, T2, and T3 (**A**) and nitrogen and potassium fertilizer treatments (**B**). Each value is expressed as the mean ± SE of 9 independent plants

**Fig. 5:**
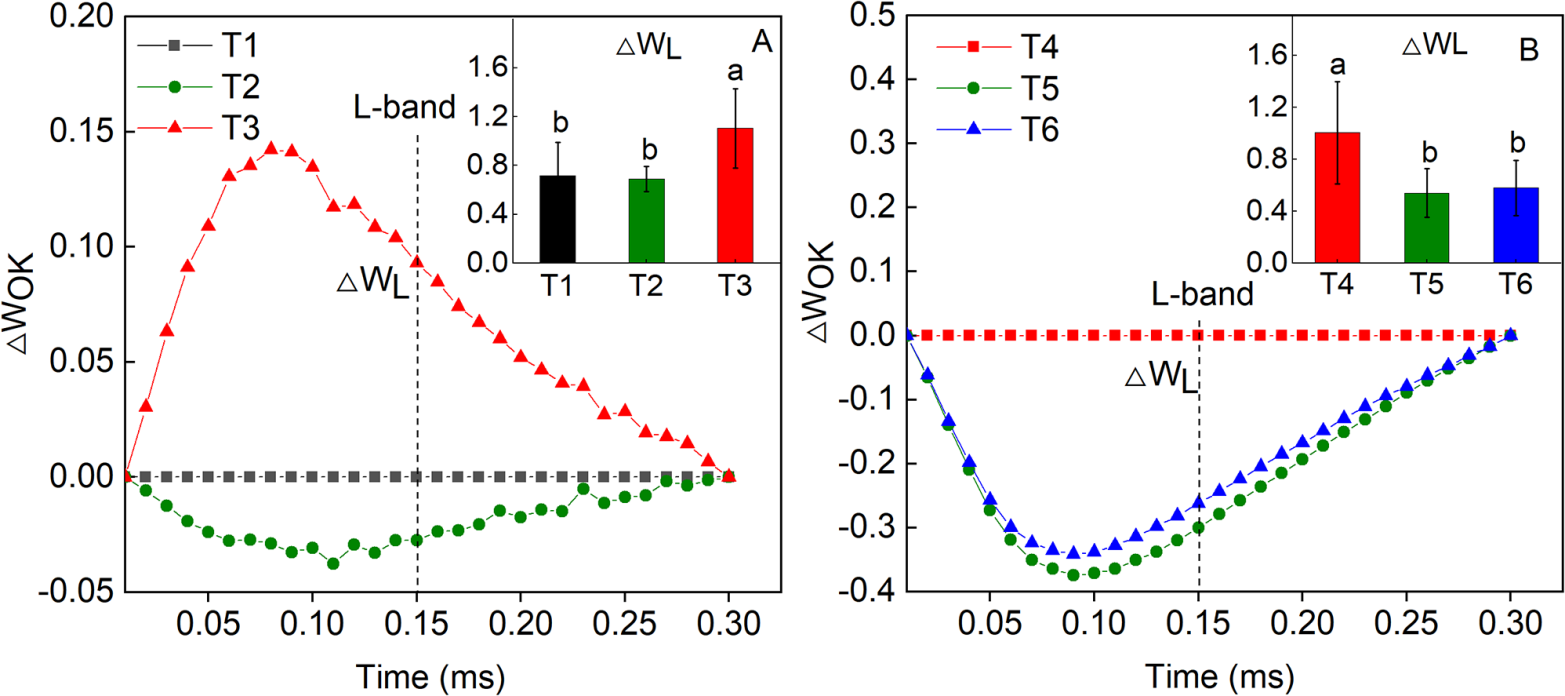
The ΔW_OK_ and ΔW_L_ of *Hosta* ‘Guacamole’ under T1, T2, and T3 (**A**) and under nitrogen and potassium fertilizer treatments (**B**). Each value is expressed as the mean ± SE of 9 independent plants. Different lowercase letters represent significant differences between treatments (*P* < 0.05)

**Fig. 6:**
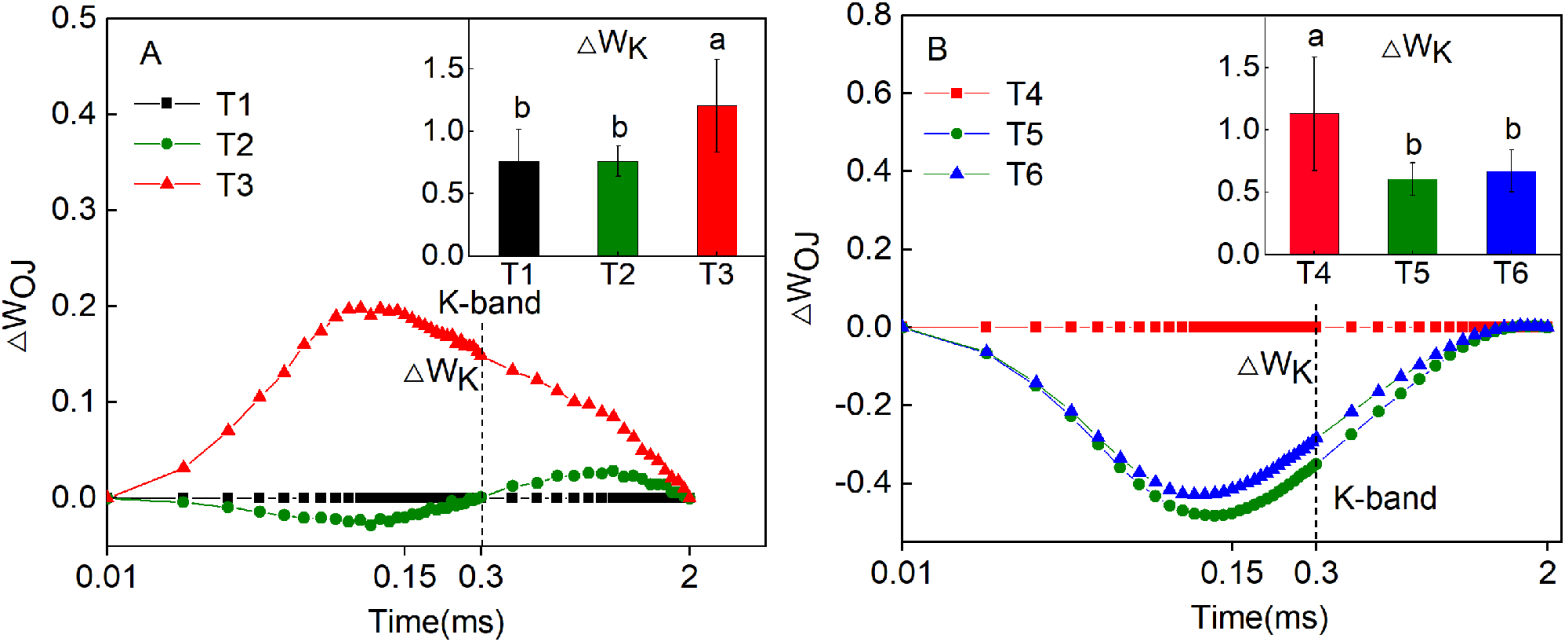
The ΔW_OJ_ andΔW_K_ in *Hosta* ‘Guacamole’ under T1, T2, and T3 (**A**) and under nitrogen and potassium fertilizer treatments (**B**). Each value is expressed as the mean ± SE of 9 independent plants. Different lowercase letters represent significant differences between treatments (*P* < 0.05)

The chlorophyll fluorescence characteristics of *Hosta* ‘Guacamole’ were further analyzed. The chlorophyll fluorescence was higher under T3 than under T1 and T2 in OJIP curve (Fig. 4A). However, it was clearly lower under T5 and T6 than under T4 in OJIP curve (Fig. 4B). In ΔW_OK_ analysis, the highest ΔW_L_ under T3 was 1.10 ± 0.324^a^ which was significantly higher than that under T1 and T2. On the other hand, it was significantly lower by 46.1% and 42.6%, respectively, under T5 and T6 than that under T4 (Fig. 5A and B). In ΔW_OJ_ analysis, ΔW_K_ was clearly higher by 58.8% and 58.6%, respectively, in T3 than that in T1 and T2 (Fig. 6A). However, after fertilizer treatments, in T5 and T6, the ΔW_K_ value was significantly lower by 46.4% and 68.2%, respectively, than that in T4 (Fig. 6B).

**Fig. 7:**
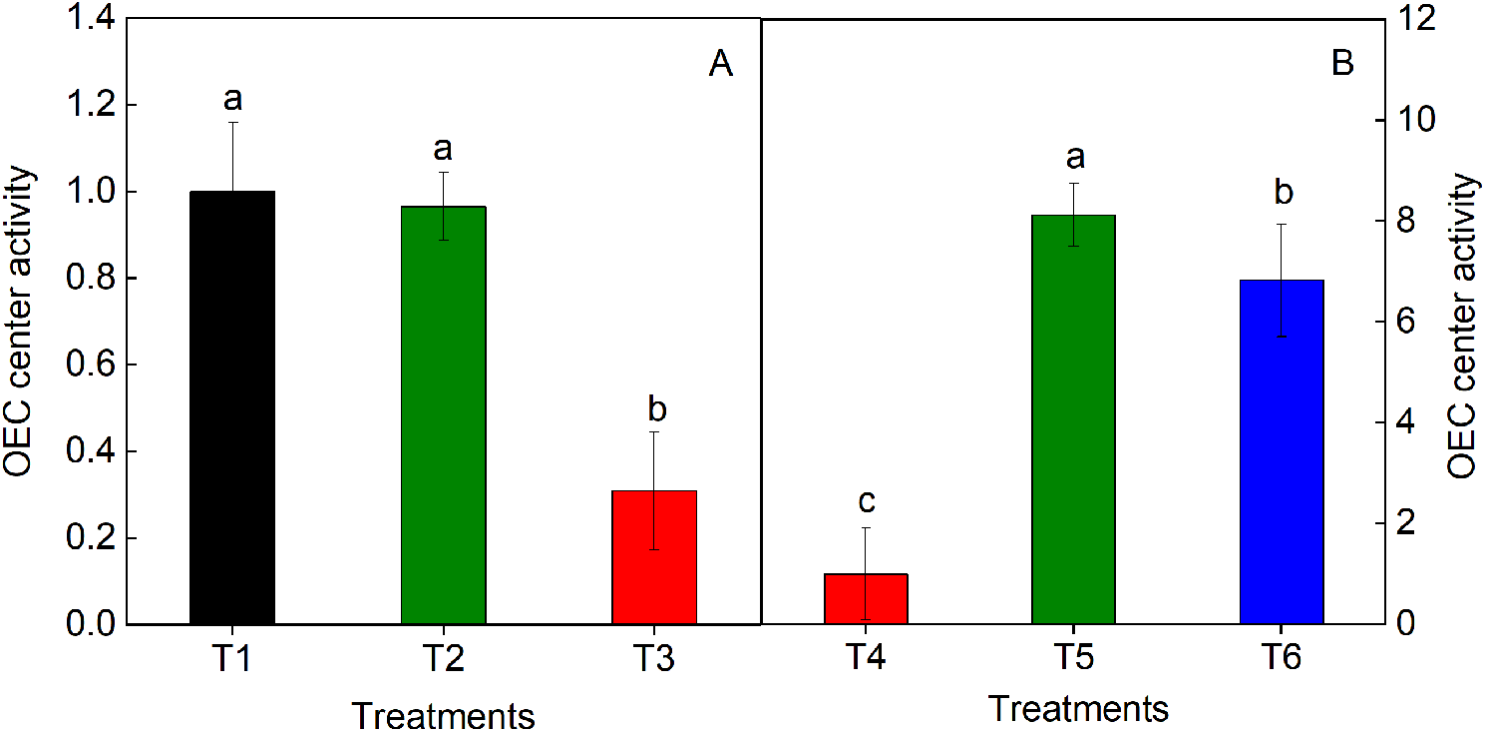
The OEC center activity under T1, T2, and T3 (**A**) and treated by nitrogen and potassium fertilizers (**B**) of *Hosta* ‘Guacamole’. Each value is expressed as the mean ± SE of 9 independent plants. Different lowercase letters represent significant differences between treatments (*P* < 0.05)

We further analyzed the oxygen-evolving complex (OEC) activity under T1–3. The OEC center activity was significantly lower in T3 than in T1 and T2. The OEC center activity was only 0.309 ± 0.136^b^; this was attributed to drought stress in T3 (Fig. 7A). However, under T5 and T6, the OEC center activity was significantly higher by 812% and 683%, respectively, than that under T4. The maximum OEC center activity was observed in T5 (Fig. 7B).

### Effect of high-nitrogen and potassium fertilizers on the JIP-test parameters in *Hosta* ‘Guacamole’ under drought stress

**Fig. 8:**
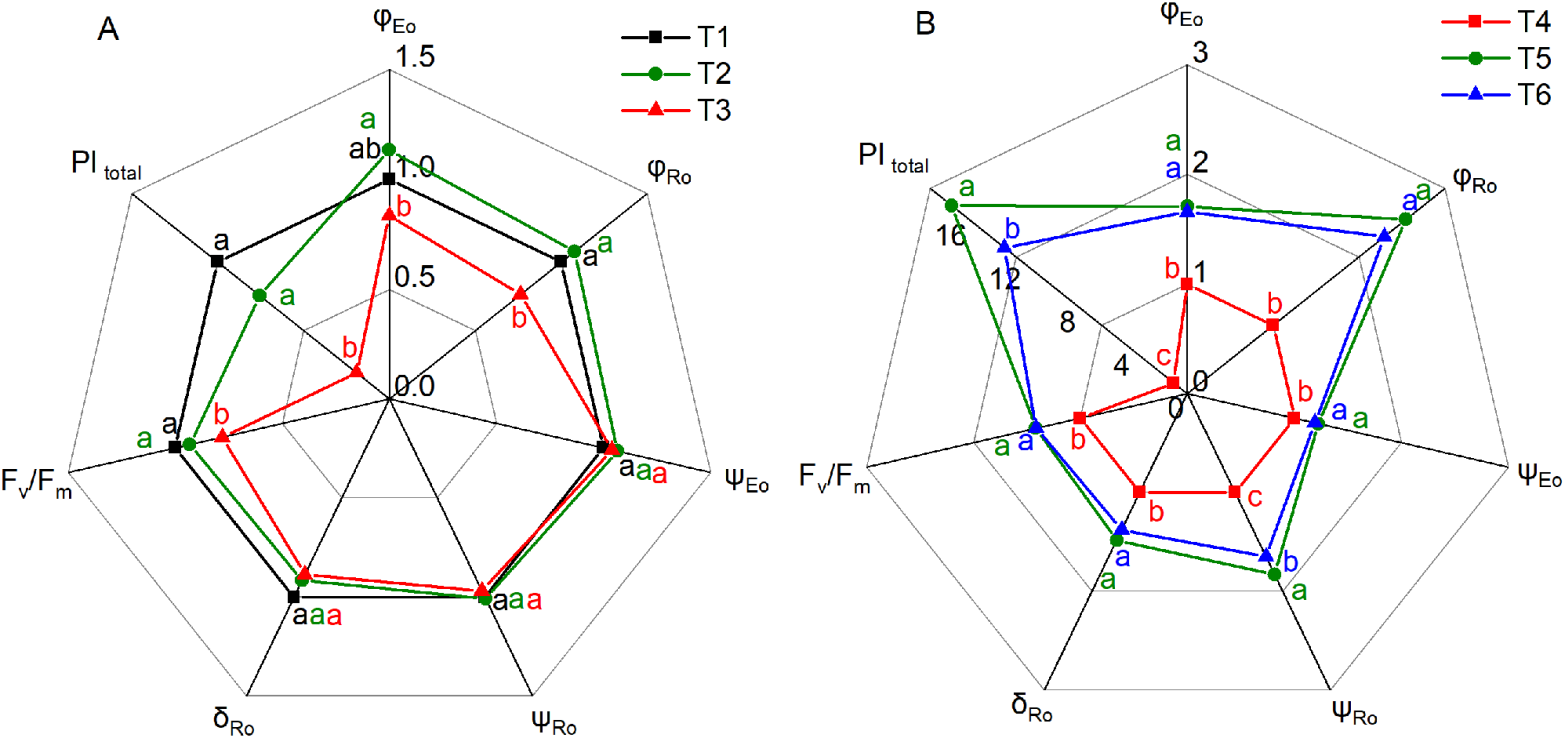
The JIP-test parameters in *Hosta* ‘Guacamole’ under T1, T2, and T3 (**A**) and under nitrogen and potassium fertilizer treatments (**B**). Each value is expressed as the mean ± SE of 9 independent plants

The JIP-test parameters of *Hosta* ‘Guacamole’ were different under various treatments (Fig. 8). The performance index for the conservation of energy from photons absorbed by the PS II antenna to the reduction of PS I acceptors (PI_total_), maximum quantum yield of primary photochemistry (F_v_/F_m_), quantum yield of the electron transport flux from Q_A_ to Q_B_ (φ_Eo_), and quantum yield of the electron transport flux until the PS I electron acceptors (φ_Ro_) were significantly lower in T3 than in T1 and T2. The PI_total_ was significantly lower by 71.6% and 64.7% in T3 than in T1 and T2, respectively. No significant differences in other JIP-test parameters including electron transport efficiency from Q_A_^−^ to the PSI electron end acceptors (ψ_Eo_), electron transport efficiency except Q_A_ (ψ_Ro_), and efficiency with which an electron from Q_B_ is transferred until PSI acceptors (δ_Ro_) were observed among the treatments (Fig. 8A). All the JIP-test parameters were higher under T5 and T6 than under T4. The maximum value of PI_total_ and ψ_Ro_ was observed under T5 treatment, which was significantly higher by 1725% and 10.7%, respectively, in T5 than in T4 (Fig. 8B).

### The alleviating effect of high-nitrogen and potassium fertilizers on the impacts of drought stress in *Hosta* ‘Guacamole’ in terms of reactive oxygen species (ROS) scavenging

**Fig. 9:**
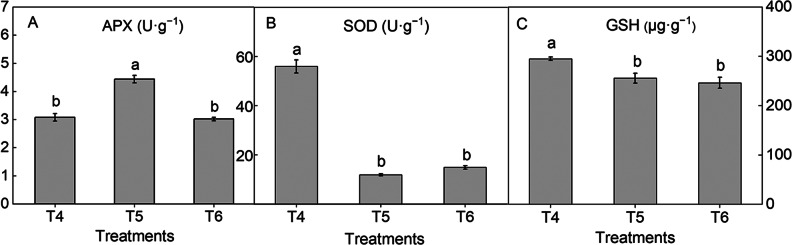
The APX (**A**), SOD (**B**), and GSH (**C**) contents under drought stress and under nitrogen and potassium fertilizer treatments. Each value is expressed as the mean ± SE of 9 independent plants. Different lowercase letters represent significant differences between treatments (*P* < 0.05)

The ROS are scavenged by ascorbate peroxidase (APX), superoxide dismutase (SOD), and non-enzymatic antioxidants such as glutathione (GSH). The APX activity was the highest in T5 (4.43 ± 0.133^a^ U·g^− 1^), which was higher by 44.1% and 46.9% than that in T4 and T6, respectively (Fig. 9A). The highest SOD activity (55.9 ± 2.68^a^ U·g^− 1^) and GSH content (294 ± 3.54^a^ µg·g^− 1^) were observed under T4. In non-enzymatic ROS scavenging system, GSH level was significantly higher by 15.3% and 19.7% in T4 than in T5 and T6, respectively. In enzymatic ROS scavenging system, the SOD activity was significantly higher by 368% and 274% in T4 than in T5 and T6, respectively (Fig. 9B, C).

## Discussion

### High-nitrogen fertilizer could avoid morphological changes of *Hosta* ‘Guacamole’ under drought stress

The frequency and severity of drought is expected to increase due to climate change [21]. Several plants are facing drought stress and high-temperature stress due to climate change; this has resulted in morphological changes including dehydrated, discolored, curled, and burned leaves [5, 6]. Thus, studying the response of *Hosta* plants to drought stress may help to design strategies for improving drought tolerance in *Hosta* ‘Guacamole’ and to efficiently deal with morphological changes due to drought stress. This study aimed to provide a basis and theoretical guidance for cultivating ornamental and horticultural plants with tolerance to drought stress. Previous studies reported that salicylic acid and sufficient nitrogen supplementation could increase leaf area under drought stress [19, 20] and efficiently improve relative water and chlorophyll contents and leaf area [8, 22]. Therefore, nitrogen fertilizer can play an important role in alleviating the effects of drought stress in plants. In this study, *Hosta* ‘Guacamole’ with water shortage (T3) exhibited growth with smaller leaf area and withering from the edge to center of leaves. However, water-soluble fertilizer, particularly high-nitrogen fertilizer, efficiently alleviated the effects of drought stress in *Hosta* ‘Guacamole’ in summer. In our results, high-nitrogen fertilizer treatment resulted in larger leaf area and greener and smoother leaves under drought stress. Furthermore, stomatal opening was smaller, transpiration rate decreased, and WUE was clearly improved, which avoided the loss of water inside the cells in drought environment. Therefore, this indicated that high-nitrogen fertilizer could avoid morphological changes under drought stress in summer.

### High-nitrogen fertilizer could improve the photosynthetic characteristics of *Hosta* ‘Guacamole’ under drought stress

Drought stress not only affects plant morphology but also changes the photosynthetic characteristics of plant leaves, which are sensitive to high temperature and drought [23]. Previous studies reported that the net photosynthesis rate, stomatic conductance, and transpiration rate were reduced in response to drought stress [24]. Similarly, our results concluded that drought stress significantly negatively affected the photosynthetic characteristics. Meanwhile, previous studies reported that salicylic acid can effectively increase photosynthesis of mustard plants under drought stress with sufficient nitrogen supplementation [20]. In our study, in the high-nitrogen treatment T5, the net photosynthetic rate and WUE were significantly enhanced and stomatal conductance and transpiration rate were decreased. T5 exhibited the highest WUE and lowest transpiration rate under drought stress, effectively enhancing the ability of drought stress resistance [22]. However, T4 (water shortage) exhibited lower *P*_n_, *G*_s_, and *T*_r_ and higher *C*_i_ than T5 and T6, which was mainly caused by stomatal closure and complex non-gassing effects [25]. In our study, high-nitrogen fertilizer could efficiently alleviate the adverse effects of drought stress in *Hosta* ‘Guacamole’ by reducing transpiration rate of leaf cells and stomatal opening size, increasing WUE, and further efficiently enhancing the photosynthetic rate.

### High-nitrogen fertilizer could improve chlorophyll fluorescence and photochemical efficiency in *Hosta* ‘Guacamole’ under drought stress

The physiological and biochemical parameters of plants under stress can be nondestructively monitored using hyperspectral imaging systems measuring chlorophyll fluorescence [8]. In our study, the ΦPS II, ETR, and qP were significantly higher in T1 than in T3, indicating that T3 resulted in photoinhibition and decreased the photochemical efficiency of *Hosta* ‘Guacamole’ plants under drought stress [26]. However, T5 and T6 alleviated the photoinhibition and improved the photochemical efficiency caused by drought stress; thus, they significantly enhanced the actual photochemical efficiency of PS II, photochemical quenching coefficient, and electron transport rate. The photochemical quenching coefficient NPQ provides insights on the level of overexcitation energy and protects photosystems from this energy by releasing heat [27]. In our study, the NPQ was significantly lower in T1 than in T2 and T3, which indicated that drought stress mainly decreased photochemical efficiency and enhanced heat-dissipation pathway in *Hosta* ‘Guacamole’. However, the NPQ was significantly lower in T5 than in T4 and T6, suggesting that high-nitrogen fertilizer efficiently alleviated photoinhibition under drought stress and enhanced the photochemical efficiency for photosystems rather than regulating heat dissipation [28].

To further understand the effect of drought stress on photosynthesis in *Hosta* ‘Guacamole’, the measurement and analysis of fast chlorophyll a fluorescence is a useful and efficient method for the assessment of many external or intrinsic adverse effects on PS II photochemistry [29]. In OJIP curve, the positive ΔL-, ΔK-, ΔJ-, and ΔI-bands appeared in T3, indicating that drought stress perhaps resulted in uncoupling of the OEC, accumulation of Q_A_^−^, or destroying the acceptor side of PS II [26]. In T3, the L-band was positive in ΔW_OK_ with bigger amplitude, and the ΔW_L_ value was significantly higher than that in T1 and T2. This indicated that T3 lost more PS II energetic connectivity, which resulted in more sensitivity to drought stress [30]. In addition, the K-band was positive in ΔW_OJ_, and the ΔW_L_ value was significantly higher in T3 than in T1 and T2, which was attributed to the damage on the OEC at the PS II donor side [31]. Furthermore, our results confirmed the possibility that the OEC activity of T3 was significantly lower than that of T1 and T2, which perhaps generated more H_2_O_2_ and destroyed cell structure [32]. However, the treatment with water-soluble high-nitrogen (T5) and high-potassium (T6) alleviated the negative effects of drought stress in the photosynthetic system. T5 and T6, particularly T5, efficiently improved PS II energetic connectivity and enhanced the OEC activity of the reaction center, which could efficiently avoid cell damage [33, 34].

Furthermore, our results revealed that the JIP-test parameters, particularly, PI _total_, F_v_/F_m_, φ_Eo_, and φ_Ro_, which quantified the conformation, structure, and function of photosynthetic apparatus, were significantly lower in T3 than in T1 and T2 treatments [30]. It indicated that drought stress resulted in the inhibition of performance index PI_total_ and quantum yield of the electron transport flux (φ_Eo_ and φ_Ro_) [31]. However, drought stress did not affect the electron transport (ψ_Eo_, ψ_Ro_, and δ_Ro_) of the photosynthetic apparatus. It indicated that the photosynthetic apparatus was different in T3, which could be attributed to the inhibition of light reactions, OEC inactivation, and reduction of chlorophyll content [31, 35]. T5 and T6 efficiently alleviated the photosynthetic apparatus stress and enhanced performance index, photosynthetic capacity, and electron transport under drought stress. T5 exhibited better alleviation and resistance effects in *Hosta* ‘Guacamole’ under drought stress and could more efficiently enhance PS II performance and PS II capacity [31]. This was attributed to the enhanced OEC activity and PS II performance by nitrogen fertilizer.

### High-nitrogen fertilizer could improve drought resistance of *Hosta* ‘Guacamole’ by stimulating enzymatic ROS scavenging system

Prolonged exposure to extreme conditions results in the increased accumulation of ROS, which easily results in lipid peroxidation and cellular damage [36]. H_2_O_2_ is an ROS that can translocate to the nucleus and acts as a signaling agent. Moreover, O_2_^−^ is very harmful and identified as the cause of photooxidative damage in plant leaves [36]. Meanwhile, OEC-depleted PS II predominantly generates H_2_O_2_ [32]. The balance of ROS in plant cells is important for plant development. The antioxidant defense system in the plant cell includes both enzymatic (e.g., SOD) and nonenzymatic (e.g., ascorbate, GSH, and α-tocopherol) antioxidants [37]. The enzymatic ROS scavenging system in plant cells includes enzymes such as SOD, POD, CAT, and APX. In the ascorbate–glutathione cycle, APX reduces H_2_O_2_ using ascorbate as an electron donor, which plays a crucial role in controlling the level of toxic byproducts of aerobic metabolism [38]. In the nonenzymatic ROS scavenging system, GSH acts as an antioxidant to reduce oxidative stress. Increased content of GSH is responsible for the reduction of oxidative stress, which plays a role in ROS detoxification, either directly or indirectly [20]. It is widely accepted that drought affects plant growth and development, changes the accumulation of compatible solutes and protective enzymes, enhances the levels of antioxidants, and inhibits energy-consuming pathways [26]. In our study, the SOD and GSH levels were significantly higher in T4 than in T5 and T6. This indicated that in *Hosta* ‘Guacamole’, the nonenazymatic ROS scavenging system was activated under drought stress to reduce damage due to ROS. However, high-nitrogen fertilizer treatment also activated enzymatic ROS scavenging system, as reflected by high APX activity and decreased ROS accumulation, which played an important role in decreasing toxic byproducts of aerobic metabolism in *Hosta* ‘Guacamole’. Our results indicated that high-nitrogen fertilizer improved drought resistance in *Hosta* ‘Guacamole’ by stimulating enzymatic ROS scavenging system. In conclusion, in summer, water-soluble fertilizer treatment to *Hosta* ‘Guacamole’ could efficiently alleviate the adverse effects of drought stress and improve drought resistance, with high-nitrogen (30-10-10).

Fertilizer exhibiting the best effect in alleviating drought stress. The drought resistance mechanism of *Hosta* ‘Guacamole’ under high-nitrogen fertilizer treatment will be further studied by studying the transcriptome and metabolome. However, we only concentrated on the effect of high-nitrogen fertilizer on *Hosta* ‘Guacamole’ under drought stress in this study, and similar experiment can be conducted on other plants in future to assess the effect of nitrogen fertilizer under drought stress.

## Conclusion

In this study, watering for once in 2 weeks in summer resulted in drought stress in *Hosta* ‘Guacamole’, causing leaf senescence, decreased chlorophyll content, limited leaf area, and reduced photosynthetic characteristics. However, high-nitrogen fertilizer treatment could efficiently alleviate and prevent the adverse effects of drought stress, enhance net photosynthetic rate and WUE, activate the activity of the reaction center OEC, enhance PS II performance and photosynthetic capacity, and reduce nonphotochemical quenching. In conclusion, the addition of 30-10-10 fertilizer to *Hosta* ‘Guacamole’ in summer could efficiently alleviate the adverse effects of drought stress and improve drought resistance. Our research provided an effective method for *Hosta* cultivation in drought season and a basis for future studies on the cultivation of *Hosta* spp. in view of climate change.

## Materials and methods

### Plant materials and growth conditions

**Fig. 10:**
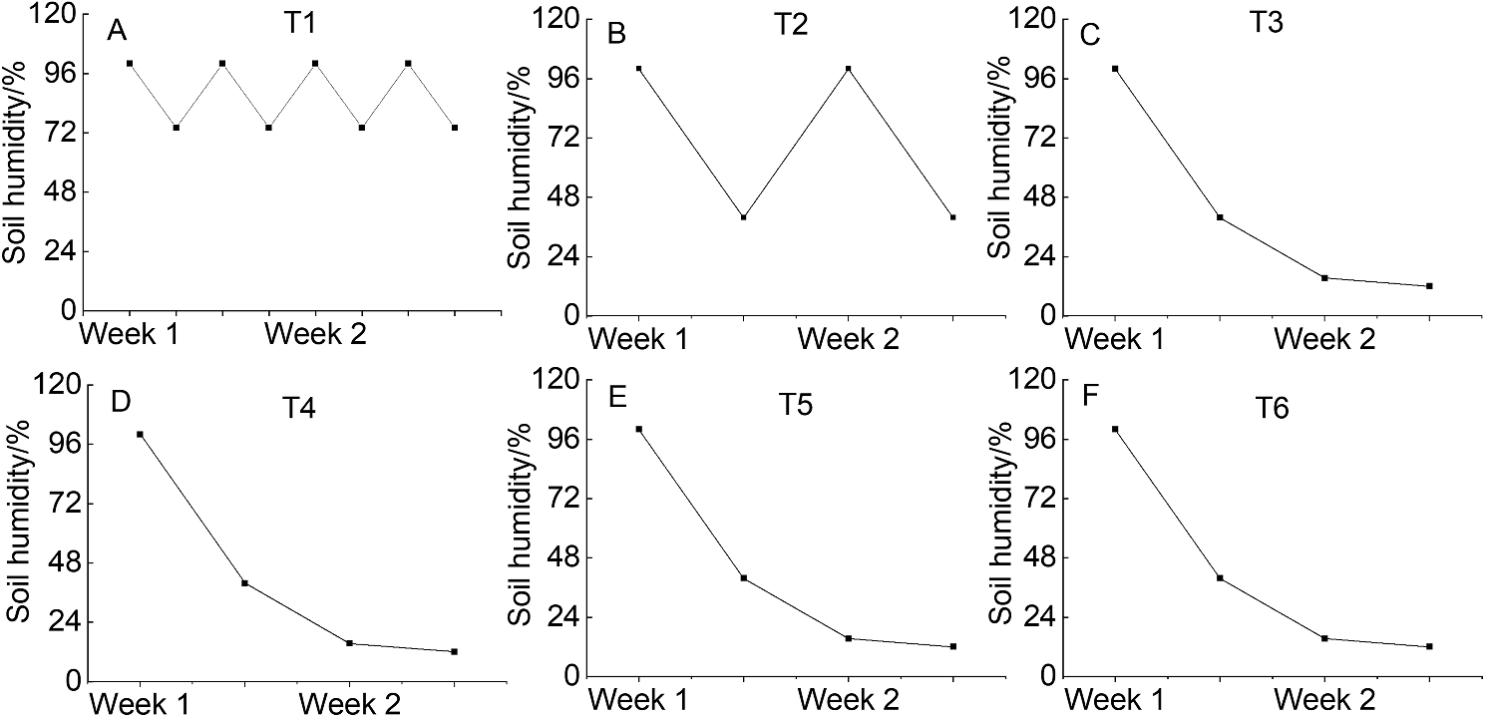
The soil humidity of T1 (**A**), T2 (**B**), T3 (**C**), T4 (**D**), T5 (**E**), and T6 (**F**)

*Hosta* ‘Guacamole’ seedlings were planted in plastic pots (diameter 12 cm) containing substrate and perlite (V:V; 3:1) and grown in a greenhouse (30°56′ N, 121°28′ E, Shanghai, China) at 30/20°C (day/night) under natural light (maximum photosynthesis photon flux density of approximately 300 µmol m^− 2^ s^− 1^) with relative humidity of 70–85%. The details of the treatments are given in Table 2. In treatments T1, T2, and T3, varying watering frequency was applied for 50 days with 30 plants in each treatment. Subsequently, the 30 plants of T3 treatment were divided into 3 groups of 10 plants. Two groups (*n* = 10 each) received T5 and T6 treatments with N:P:K 30-10-10 and 15-10-30 fertilizers (ANOREL, Billy Fu Horticulture (Shanghai) Co., Ltd. ), respectively, and the remaining group (*n* = 10) received no fertilizer treatment (T4) for 30 days. The electric conductivity of fertilizers was 500 µs cm^− 1^, and the soil humidity in different treatment was different. The soil humidity was determined using hand-held soil tachymeter (Tianjin Tianhang Zhiyuan Technology Co., LTD, China) four times a week at fixed time (Fig. 10). Soil humidity was determined using 10 plots each time.

**Table 2:** Various watering and fertilizer treatments

Treatments	Condition
Treatment 1 (T1)	watering for twice a week for 50 days (*n* = 30)
Treatment 2 (T2)	watering once a week for 50 days (*n* = 30)
Treatment 3 (T3)	watering once in 2 weeks for 50 days (*n* = 30)
Treatment 4 (T4)	continue watering once in 2 weeks with 10 plants from T3
Treatment 5 (T5)	watering with 30-10-10 N-P-K fertilizer once in 2 weeks with 10 plants from T3
Treatment 6 (T6)	watering with 15-10-30 N-P-K fertilizer once in 2 weeks with 10 plants from T3

### Measurements of area and chlorophyll content in the leaves of *Hosta* ‘Guacamole’

To determine the growth of leaves, a leaf area meter (Yaxin-1241, Beijing Yaxin Liyi Technology Co., Ltd., Beijing, China) was used. Chlorophyll contents were determined using a portable chlorophyll meter SPAD-502 PLUS (Konica Minolta Optics, Tokyo, Japan) as per the method by Iqbal et al. [20]. To further analyze the effect of drought stress and water-soluble fertilizers on the leaf color, chroma meter (CR-400, KONICA MINOLTA, Japan) was used to measure the chromatic aberration values L^*^, a^*^, and b^*^ that denote the brightness value, red-green degree value, and yellow-blue degree value, respectively. The leaf area, chlorophyll content, and chromatic aberration were measured using 15 plants in T1, T2, and T3 treatments. After applying the fertilizers at the 30th day, these parameters were measured using 10 plants each from T4, T5, and T6 treatments.

### Assessment of photosynthetic parameters and chlorophyll fluorescence

The photosynthetic parameters, including *P*_n_, *g*_*s*_, *T*_r_, *C*_i_, WUE, and VPD, were measured using CIRAS-3 portable photosynthesis system (PP Systems, Amesbury, MA, USA) with 260 µmol m^− 2^ s^− 1^ PPFD, 60–70% relative humidity, and ambient CO_2_ of 390 ppm. The measurements were performed using 9 plants from T1, T2, T3, T4, T5, and T6 treatments, respectively. Chlorophyll fluorescence of the leaves was measured using IMAGING-PAM (MAXI) (Zealquest Scientific Technology Co., Ltd., Shanghai, China). The leaves were placed in dark for 30 min prior to measurement. Maximum quantum yield of PS II (F_v_/F_m_), actual photochemical efficiency of PS II (ΦPS II), photochemical quenching coefficient (qP), non-photochemical quenching coefficient (NPQ), and electron transport rate (ETR) were measured. These measurements were performed thrice on four points on each side of the main vein from the tip to middle of the leaves.

### Chlorophyll fluorescence rise kinetics and JIP-test parameters

Chlorophyll fluorescence rise kinetics were assessed using a Handy PEA continuous excitation fluorimeter (Handy Plant Efficiency Analyzer; Hansatech Instruments, Ltd., King’s Lynn, UK). The relative fluorescence parameters were calculated by double normalization of the moment chlorophyll fluorescence values to the end point within different intervals with the OJIP part of the transient-OP, OK, OJ, and OI. Following formulae were used for the calculations to clarify the structure and function of the photosynthetic apparatus by describing the primary photosynthetic reactions in PS II [29, 39]. The fluorescence parameters are listed in Table 3. Other formulae are given as follows: ΔW_OK_ = W_OK (treatment)_ − W_OK (control)_; ΔW_OJ_ = W_OJ (treatment)_ − W_OJ (control)_; ΔW_L_ =W_L(treatment)_ − W_L(control)_; and ΔW_K_ =W_K(treatment)_ − W_K(control)_. The JIP-test parameters including PI _total_, φ_Eo_, F_v_/F_m_, φ_Ro_, δ_Ro_, ψ_Ro_, and ψ_Eo_ were measured with nine replications using Handy PEA continuous excitation fluorimeter for each treatment.

**Table 3:** The fluorescence parameters

Fluorescence parameters	Explanation
V_t_ = (F_t_ − F_o_)/(F_m_ − F_o_)	Relative variable fluorescence at time t
W_OJ_ = (F_t_ − F_o_)/(F_J_ − F_o_)	Ratio of variable fluorescence F_t_ − F_O_ to the amplitude F_J_ − F_O_
W_OK_ = (F_t_ − F_o_)/(F_K_ − F_o_)	Ratio of variable fluorescence F_t_ − F_O_ to the amplitude F_K_ − F_O_
PI_total_	Performance index for the conservation of energy from photons absorbed by the PS II antenna to the reduction of PSI acceptors
φ_Eo_	Quantum yield of the electron transport flux from Q_A_ to Q_B_
F_v_/F_m_	Maximum quantum yield of primary photochemistry
φ_Ro_	Quantum yield of the electron transport flux until the PSI electron acceptors
δ_Ro_	Efficiency with which an electron from Q_B_ is transferred until PSI acceptors
ψ_Ro_	Electron transport efficiency except Q_A_
ψ_Eo_	Electron transport efficiency from Q_A_^−^ to the PSI electron end acceptors
OEC activity	oxygen-evolving complex activity; OEC centers = [1 − (V_K_/V_J_)] _treatment_ / [1 − (V_K_/V_J_)] _control_

### ROS scavenging

ROS including superoxide anion radical and H_2_O_2_ are scavenged by SOD and APX, respectively [36]. The SOD activity, APX activity, and GSH contents were measured as per the method by Yang et al. [37] using the kits by Wuhan ProNets Biotechnology Co, Ltd (Wuhan, China). The leaves of three individuals were mixed and considered as one replication. The experiment included three replicates. The details of extraction and determination of SOD activity, APX activity, and GSH contents are given in supplementary S1.

### Statistical analysis

Statistical analysis was performed using SPSS 19.0 (IBM, Inc., Armonk, NY, USA). The morphological and physiological parameters were analyzed using Duncan’s test (*P* < 0.05). The data were processed using Microsoft Excel 2007 (Redmond, CA, USA) and plotted using Origin Pro Version 8.5E (OriginLab, Northampton, MA, USA).

### Electronic supplementary material

Below is the link to the electronic supplementary material.

Supplementary Material 1

## Data Availability

All data generated or analyzed during this study are included in this published article.

## Abbreviations

APX: ascorbate peroxidase
*C*_i_: intercellular CO_2_ concentrations
ETR: electron transport rate
F_v_/F_m_: Maximum quantum yield of PS II
*g*_*s*_: stomatal conductance
GSH: Glutathione
NPQ: non-photochemical quenching coefficient
*P*_n_: net photosynthetic rate
qP: photochemical quenching coefficient
SOD: superoxide dismutase
*T*_r_: transpiration rate
VPD: vapor pressure deficit
WUE: water use efficiency
ΦPS II: actual photochemical efficiency of PS II
φ_Ro_: quantum yield of the electron transport flux until the PSI electron acceptors
δ_Ro_: efficiency with which an electron from Q_B_ is transferred until PSI acceptors
ψ_Ro_: electron transport efficiency except Q_A_
ψ_Eo_: electron transport efficiency from Q_A_^−^ to the PSI electron end acceptors
