# Supplementary material for: High-nitrogen fertilizer alleviated adverse effects of drought stress on the growth and photosynthetic characteristics of Hosta ‘Guacamole’
Source: BMC Plant Biol. 2024 Apr 18;24:299. doi: 10.1186/s12870-024-04929-5 (PMC11025241; doi:10.1186/s12870-024-04929-5)
Supplement: Supplementary file 1 — Supplementary Material 1 [file 12870_2024_4929_MOESM1_ESM.docx]

**Supplementary 1**

- 1. **SOD method:**

After grinding the sample into powder with liquid nitrogen, weigh about 0.1g of the sample, add 0.4mL of the extract, and make the ice bath homogenization; Centrifuge at 4℃ at 8000g for 10min, take the supernatant and put it on the ice to be measured; Take 18µL supernatant and add 48 µL extract, 12µL 0.037U/L xanthine oxidase, 36µL 1.25mmol/l nitrotetrazolium blue chloride (NBT) solution, 80µL distilled water and 6μL75mmol/ l xanthine solution in sequence to the 96-well plate, thoroughly mix, and bathe at 37℃ for 30min. The absorption value of each tube was measured at 560nm and was denoted as A measurement. Calculation of inhibition percentage: P (inhibition percentage) = (ΔA_CK_ -ΔA _determination_) ÷ ΔA_CK_ ×100%;

SOD activity (U/g) = 1.11× P ÷(1- P)÷W×F×V _sample total_ (V _sample total_: Add extraction liquid volume, 0.5mL; W: Sample mass, g; F: Sample dilution ratio);

**1.2 APX method:**

The 0.15g sample was weighed and added to 1mL 50mmol/LPBS for ice bath homogenization. Centrifuge at 13000g4℃ for 20min, take the supernatant and put it on ice to be measured; 20µL supernatant, 140µL50mmol/LPBS, 20µL0.4mmol/LVC solution, and 20µL1mmol/LH_2_O_2_ were added to the 96-well plate, quickly mixed, and the absorption value A10s at 290nm and A130s at 130s were recorded. Calculate ΔA = A_10s_-A_130s_;

APX(U/g) =3.57×ΔA÷W (W: Sample mass, g);

**1.3 GSH method:**

Weigh about 0.15g sample, add 0.8ml extract, ice bath homogenate, 8000g, centrifuge at 4℃ for 10min; Take the supernatant and place it at 4℃ to be measured. Take 20μL supernatant, add 140μL reagent 1 and 40μL reagent 2 successively, mix well, and let stand for 2min to detect absorbance A at 412nm.

GSH content (μg/g) =C÷W×V _sample total_

C: The glutathione concentration (mg/mL) was obtained by placing the A determination into the standard curve; V _sample total_: total volume of extract, 0.8mL; W: Sample mass, g.
